# Comparison of linkage analysis methods for genome-wide scanning of extended pedigrees, with application to the TG/HDL-C ratio in the Framingham Heart Study

**DOI:** 10.1186/1471-2156-4-S1-S93

**Published:** 2003-12-31

**Authors:** Benjamin D Horne, Alka Malhotra, Nicola J Camp

**Affiliations:** 1 Genetic Epidemiology, Department of Medical Informatics, University of Utah School of Medicine, Salt Lake City, Utah, USA; 2 Cardiovascular Department, LDS Hospital, Intermountain Health Care, Salt Lake City, Utah, USA; 3 Department of Human Genetics, University of Utah School of Medicine, Salt Lake City, Utah, USA

## Abstract

**Background:**

High triglycerides (TG) and low high-density lipoprotein cholesterol (HDL-C) jointly increase coronary disease risk. We performed linkage analysis for TG/HDL-C ratio in the Framingham Heart Study data as a quantitative trait, using methods implemented in LINKAGE, GENEHUNTER (GH), MCLINK, and SOLAR. Results were compared to each other and to those from a previous evaluation using SOLAR for TG/HDL-C ratio on this sample. We also investigated linked pedigrees in each region using by-pedigree analysis.

**Results:**

Fourteen regions with at least suggestive linkage evidence were identified, including some that may increase and some that may decrease coronary risk. Ten of the 14 regions were identified by more than one analysis, and several of these regions were not previously detected. The best regions identified for each method were on chromosomes 2 (LOD = 2.29, MCLINK), 5 (LOD = 2.65, GH), 7 (LOD = 2.67, SOLAR), and 22 (LOD = 3.37, LINKAGE). By-pedigree multi-point LOD values in MCLINK showed linked pedigrees for all five regions, ranging from 3 linked pedigrees (chromosome 5) to 14 linked pedigrees (chromosome 7), and suggested localizations of between 9 cM and 27 cM in size.

**Conclusion:**

Reasonable concordance was found across analysis methods. No single method identified all regions, either by full sample LOD or with by-pedigree analysis. Concordance across methods appeared better at the pedigree level, with many regions showing by-pedigree support in MCLINK when no evidence was observed in the full sample. Thus, investigating by-pedigree linkage evidence may provide a useful tool for evaluating linkage regions.

## Background

Obesity, diabetes, and hypertension are closely associated with low levels of high-density lipoprotein cholesterol (HDL-C) and elevated levels of triglycerides (TG), and are recognized as jointly increasing coronary risk [1]. These factors are the major components of the metabolic syndrome as outlined in the report of the National Cholesterol Education Program's Adult Treatment Panel (ATP) III [2].

In an evaluation of a genetic component of the metabolic syndrome, Shearman et al. [3] reported a genome-wide scan for loci linked to TG/HDL-C ratio using Framingham Heart Study (FHS) family data and SOLAR [4], which performs variance-components analysis. SOLAR allows for pedigrees of arbitrary size by estimating multi-point identity by descent (IBD) probabilities from the exact two-point IBDs, which are then used in variance components linkage statistic calculations. SOLAR assumes multivariate normality, but is considered model-free and relies on little prior knowledge of the underlying genetic model.

In contrast, parametric linkage analysis requires specification of the underlying model of genetic inheritance. These models are usually unknown and must be estimated. Commingling analysis can be used to estimate genetic parameters from the phenotypic data. Although parametric analysis requires model specification, it can provide statistical power beyond that of model-free analyses [5]. Three linkage software packages able to perform parametric analyses include LINKAGE [6], GENEHUNTER [7], and MCLINK [8].

LINKAGE calculates exact two-point IBD probabilities for use in two-point linkage analysis, and can be used for pedigrees of arbitrary size. Two-point analysis is less sensitive to misspecification of the model parameters than multi-point, since these are absorbed into the maximization over the recombination fraction, θ. For a detailed description see Göring and Terwilliger [9]. Two-point analysis, however, can be sensitive to false positives, due to misspecification of marker allele frequencies or rare alleles segregating in some families, or have low power, due to poor IBD probability estimates.

Parametric analysis in GENEHUNTER (GH) also is an exact likelihood method. Multi-point LOD analysis that is less sensitive to inaccurate allele frequencies and is superior at determining IBD probabilities can be calculated; however, multi-point LOD statistics are constrained for θ = 0 and thus can be prone to false-negative results (loss of power) if the model parameters are misspecified. Further, the pedigree size capacity for GH is limited and with large pedigrees can require extensive trimming, which can lead to loss in power due to the elimination of important genealogical and segregational information.

MCLINK is a Markov chain Monte Carlo (MCMC) method that uses blocked Gibbs sampling to estimate multi-point IBD probabilities on extended pedigree structures. In addition, MCLINK supports a robust multi-point theta-LOD (TLOD) statistic [9,10]. The TLOD is a hybrid-multi-point statistic that uses multi-point IBD probabilities estimated from all available marker data, but calculates the LOD statistic under a two-point paradigm, thus combining the benefits of the two-point analysis without losing haplotype information.

Thus, while multi-point analysis methods have become popular, exact multi-point methods cannot evaluate large, extended pedigrees as exact two-point methods can. Estimation methods may circumvent these issues, but may have their own weaknesses. The major objective of this study was to compare the linkage analysis results of LINKAGE, GH, and SOLAR to a potentially more robust parametric method, MCLINK, that incorporates all available pedigree information into the linkage analysis.

## Methods

### Phenotype

The data for this study consisted of real FHS data for both the original and offspring cohorts, as provided in Problem 1 of the Genetic Analysis Workshop 13 (GAW13) data set. As noted by Shearman et al. [3], cholesterol measurements were made on 12–14 h fasting blood samples. TG concentrations were measured only once for FHS original cohort participants, at Exams 10, 11, or 12. HDL cholesterol measurements from the same exam as these TG levels were used to compute the TG/HDL-C ratio. For the offspring cohort, TG and HDL-C were measured at each of the first five study cycles. The TG/HDL-C ratio was calculated for each of these cycles and the lowest ratio was selected as the one to be used in the study. The rationale was that the lowest value would generally represent measurements that were less encumbered by environmental factors. Because cholesterol values tend to become more extreme with age, this process resulted in essentially all TG/HDL-C values being drawn from offspring study cycle 1 or 2; thus the time frame of the cholesterol measurements was the early 1970s for both original and offspring cohorts. The TG/HDL-C ratio was normalized using the natural logarithm to reduce skewing of the distribution.

Prior to linkage analysis, the TG/HDL-C ratio was adjusted by regression for age, body mass index (BMI), systolic blood pressure (SBP), and glucose level because of the definition of the metabolic syndrome in the ATP III guidelines [2], with BMI serving as an obesity-related surrogate for waist circumference. The same or nearest exam for which these measures were available was used in the analyses. The regression residuals were taken to represent the TG/HDL-C ratio free from the effects of the other factors (correlation of TG/HDL-C and these residuals was r = 0.80). Adjustment for smoking and alcohol was not used because they are not components of the metabolic syndrome and they had essentially no effect on the TG/HDL-C ratio (correlation of residuals before and after adjustment for smoking and alcohol was very high, r = 0.996). Physical activity and estrogen replacement therapy were not available in the GAW13 FHS data set and thus could not be controlled as before [3]. Further, lipid-lowering medication use was not available in the GAW13 data set. Out of the 2885 FHS participants in the 330 available pedigrees of the genome scan, 2467 participants had an evaluable TG/HDL-C ratio and 2,461 had measured values for all factors.

### Genetic model

We used commingling analysis to determine genetic parameters assuming normal distributions under a major gene model. Commingling analysis revealed significant evidence for two populations with mean TG/HDL-C residuals of 1.26 and -0.08, with common standard deviation 0.56. Gene frequencies of 0.01 and 0.1 were used for the dominant and recessive models. Four parametric models (HighDom, HighRec, LowDom, and LowRec) were analyzed as we were interested in locating susceptibility genes for both low (protective) and high (risk) TG/HDL-C ratios. All LOD and TLOD scores were maximized over the proportion of families linked using heterogeneity LODs (α).

### By-pedigree support

We used by-pedigree classical multi-point LOD statistics (with θ = 0) available from MCLINK to assess if a pedigree was linked to a region. Each region was defined as the 20 cM surrounding the peak full sample LOD. A pedigree was considered linked to that region if a pedigree LOD > 0.588 was observed, which corresponds to a nominal *p*-value of 0.05. The region size was estimated using the 1-LOD drop from the peak of the summed pedigree LODs (sumLOD) for linked pedigrees only, and also from the observed recombinants. We used the pedigree multi-point graphs to estimate positions of recombinants, and used a drop of 0.5 LOD units to indicate the existence of a recombinant.

## Results

### Full sample linkage analyses

General characteristics of the study population are given in Table 1. Overall, 14 regions were found to have at least suggestive linkage with LOD scores above 1.9 [11] by at least one analysis (Table 2).

For the parametric analyses, regions were identified under both the high and low TG/HDL-C ratio models. Analyses evaluating potential loci predisposing to a high TG/HDL-C ratio identified five potential regions on chromosomes 1, 3, 5, and 7 (Table 2). The highest scores (2.34 and 2.65) were on chromosome 5 at 103 cM and 125 cM, both identified using GH with a dominant model. The locus at 125 cM was located by all three parametric methods, the second, at 103 cM, was also located using MCLINK.

Parametric analyses to locate predisposition loci for low TG/HDL-C ratio, which could indicate a genetically protective effect, identified seven regions on chromosomes 2, 8, 10, 13, 17, 19, and 22 (Table 2). The best two-point score from LINKAGE was 3.37 on chromosome 22 (21 cM, recessive model). This locus was not located by other parametric methods, but was identified by SOLAR two-point analysis. The highest multi-point score was 2.29 on chromosome 2 (210–17 cM, recessive model) and was identified by MCLINK. This locus was also located by LINKAGE (1.26 at 205 cM).

Model-free variance components analysis identified two further regions on chromosomes 3 and 7 (Table 2). These regions were both also located by MCLINK, and GH located the region on chromosome 7 at approximately 160 cM.

### By-pedigree linkage evidence

We followed up the best regions for each analysis method on chromosomes 2 (201 cM, MCLINK), 5 (125 cM, GH), 7 (163 cM, SOLAR), and 22 (21 cM, LINKAGE) using multi-point by-pedigree support as provided by MCLINK. Despite using only MCLINK to identify linked pedigrees, at least three linked pedigrees (LOD > 0.588) were found for each region. For each of the regions analyzed, no conflicting recombinants were evident. Table 3 illustrates the characteristics of the regions, including the number of linked pedigrees found for each region, the peak sumLOD (sum of the multi-point LODs for the linked pedigrees) and the region size and boundaries both as estimated by the 1-LOD drop and from inspection of estimated recombinants; in addition, the number of pedigrees that define the left and right recombinant boundaries are given. The smallest region (9 cM) was on chromosome 7, using recombinant boundaries, however only one recombinant on each side defined this region. The best recombinant evidence was for the region on chromosome 2 where five recombinants (two on the left hand side and three on the right) defined an 18-cM region. In addition, this region obtained the highest sumLOD (7.23), although this defined the largest 1-LOD drop (21 cM).

## Conclusions

Several loci were found by multiple analysis methods, including a region on chromosome 17 identified by all methods. This is reassuring, although a large range of LODs was often seen for the same locus. Across all 14 regions identified, only four loci were identified using only a single analysis method (one LINKAGE, two GH, and one MCLINK), six regions were identified with two methods, three regions were identified with three methods, and one region on chromosome 17 at 126–129 cM was located by all four methods, although only GH identified the region with at least suggestive evidence. This region on chromosome 17 was also the best region observed by Klos et al. [12] for a related total cholesterol/HDL ratio phenotype (LOD: 2.48), although Klos et al. did not have any other TG- or HDL-C-related phenotypes with LOD > 1.9.

In the analyses here, no single method identified all the regions. MCLINK performed the best in this regard with LODs > 1.0 in 10 of the total 14 regions, and 3 of the 4 regions missed were those identified by only a single method.

Shearman et al. [3], using SOLAR to analyze a related TG/HDL-C ratio phenotype, reported one region with suggestive evidence for linkage on chromosome 7 at 155 cM (LOD = 2.5), and a further four regions with LOD > 1.0 on chromosomes 3 (LOD 1.8 at 140 cM), 11 (LOD 1.1 at 125 cM), 16 (LOD 1.1 at 75 cM), and 20 (LOD 1.7 at 35 cM). Our SOLAR results similarly identified regions on chromosomes 3 and 7 with comparable magnitude (Table 2).

In general, better agreement was found in this study between the parametric methods than with the results from SOLAR, and in particular between LINKAGE and MCLINK. This may be due to the similarity in pedigree structures analyzed. In addition, MCLINK agreed better with GH than it did LINKAGE. The better agreement of GH with MCLINK may be due to the multi-point nature of both analyses. Further, MCLINK obtained better agreement with SOLAR than the other two parametric methods. However, our model-free SOLAR results were more concordant with those of Shearman et al. [3] than with the parametric findings.

Even if a much less stringent LOD threshold were imposed on the analyses of these data, no single method used in this study would have identified all 14 regions here. MCLINK identified only moderate-size peaks compared across parametric methods, but located evidence for linkage (LOD > 1.0) for 10 of the total 14 regions, and had the best concordance with each of the other methods, LINKAGE located 8 out of 14 regions, GH 7 out of the total 14, and SOLAR only 4. Of the 7 locations it found, GH found them all with LOD > 1.5. The lower number located by GH may be partly due to loss of pedigree structure.

The best four regions identified by each method were followed up with by-pedigree analysis using MCLINK multi-point LODs. Despite the lack of highly confirmatory full sample scores, the by-pedigree support identified at each locus was good (at least three pedigrees linked to each region). This may indicate better consistency of LOD findings at the pedigree level.

Other evaluations of linkage for TG and HDL-C phenotypes have been published, in addition to those of Shearman et al. [3] and Klos et al. [12] that are mentioned above, although only Shearman et al. have addressed the ratio of TG/HDL-C. This study replicated a finding by Peacock et al. [13] for HDL-C on chromosome 13 at 27.54 cM (LOD: 2.36), although it did not replicate their other suggestive finding on chromosome 5 (39.89 cM, LOD: 3.64). Pajunkanta et al. [14] reported a region on chromosome 2 at 186 cM for TG among Finnish families, but their other region on chromosome 10 was not replicated by this study. Among that Finnish population, Soro et al. [15] later reported on HDL-C, but none of their regions coincided with the current study's findings. A study of both HDL-C and TG as separate measures by Coon et al. [16] also had no coincident findings.

The generally poor concordance between this study's findings and the results of studies other than Shearman et al. [3] may be due to the use of the ratio of TG and HDL-C, which is regarded as a marker of insulin resistance or the metabolic syndrome, and not just elevated cholesterol. The current study evaluated a larger population with more families than most other studies, though, and also examined loci for low TG/HDL-C ratio as a potentially protective factor, whereas other studies have only addressed loci linked to increased risk lipids (elevated TG or reduced HDL-C).

This study has identified loci linked to TG/HDL-C ratio that may relate to the metabolic syndrome or insulin resistance, including some that may increase coronary risk and some that decrease risk. Further, it indicates that multi-point and two-point analyses, based on exact inheritance probabilities (LINKAGE, GH) and estimation methods (SOLAR, MCLINK) IBD, and parametric (LINKAGE, GH, MCLINK) and model-free (SOLAR) analyses can all contribute to the identification of loci for complex quantitative traits. Although we attempted no formal comparison using simulations that would enable definitive conclusions about the comparison of methods, these results do provide a valuable practical comparison of approaches. We found reasonable consistency across multiple methods, and suggest that consistency of findings, in addition to LOD peak size, could be used in identifying potential regions. Our results indicate that the MCMC method, MCLINK, behaves as one might expect for a method that has parallels with both two-point full pedigree analyses and limited pedigree structure multi-point analyses, with reasonable correspondence with both methods. Our results further suggest the utility of inspecting by-pedigree support for regions, both to increase confidence and to increase region definition and narrowing.

## Figures and Tables

**Table 1:** Population characteristics for the GAW13 FHS Cohorts.

**Characteristic**	**Overall**	**Original**	**Offspring**
Age (years)	46.0 ± 14.8	59.8 ± 7.4	39.4 ± 12.7
Sex (male)	48.5%	46.7%	49.4%
TG/HDL-C ratio	1.8 ± 2.0	3.0 ± 2.7	1.3 ± 1.2
Systolic blood pressure (mm Hg)	126 ± 20	138 ± 21	120 ± 16
Body mass index (kg/m^2^)	26 ± 4	27 ± 4	25 ± 4
Glucose (mg/dL)	98 ± 22	98 ± 30	98 ± 16

**Table 2:** Regions with at least suggestive evidence under one analysis.^A^

**Chr**	**cM**	**Nearest Marker**	**LINKAGE**	**GH**	**MCLINK**^B^	**SOLAR**^C^
1	276	203yg9	-^D^	-	1.93 (276 cM, HD)	-
2	201–05	GATA65C03	1.26 (205 cM, lr)	-	2.29 (201 cM, lr)	-
3	135–41	GATA84B12	-	-	1.74 (135 cM, HD)	1.89 (m;141 cM)
						2.50 (2;135 cM)
3	194	GATA3H01	1.95 (195 cM, Hr)	-	1.98 (194 cM, Hr)	-
5	103	GATA69H12	-	2.34 (103 cM, HD)	1.51 (103 cM, HD)	-
5	125	ATA4D10	1.45 (125 cM, HD)	2.65 (125 cM, HD)	1.57 (125 cM, HD)	-
7	48–57	GATA13G11	-	1.97 (52 cM, HD)	-	-
7	161–69	GATA32C12	-	1.52 (161 cM, Hr)	1.17 (156 cM, HD)	2.12 (m;169 cM)
						2.67 (2;163 cM)
8	176	UT721	-	2.26 (176 cM, lr)	-	-
10	32–33	ATA31G11	1.82 (32 cM, lD)	-	1.94 (33 cM, lr)	-
13	36	GATA86H01	2.12 (36 cM, lD)	-	-	-
17	126–29	044xg3	1.12 (128 cM, lr)	2.10 (129 cM, lr)	1.44 (126 cM, lr)	1.13 (2;127 cM)
19	0–11	GATA44F10	1.63 (11 cM, lD)	2.07 (0 cM, lD)	1.61 (0 cM, lD)	-
22	21	Mfd313	3.37 (21 cM, lr)	-	-	1.28 (2; 21 cM)

^A^A region is reported only when at least one analysis produced a score over 1.9. In those regions all other analyses that reached at least a score >1.0 are also reported. ^B^H, loci linked to high TG/HDL ratio; l, low TG/HDL-C ratio; D, dominant model; r, recessive model. ^C^Both two- and multi-point variance component analyses were performed in SOLAR (2 indicates two-point, m indicates multipoint results). ^D^Indicates no score above 1.0.

**Table 3:** Regions with at least suggestive evidence under one analysis.^A^

	**LINKAGE**	**GH**	**MCLINK**	**SOLAR**
Chromosome	22	5	2	7
Full Sample LOD	3.37 (2pt)	2.65 (mpt)	2.29 (mpt)	2.67 (2pt)
cM	21 cM	125 cM	201 cM	163 cM
No. peds linked	9	3	11	5
sumLOD	6.28 (mpt)	3.16 (mpt)	7.23 (mpt)	3.01 (mpt)
1-LOD size	13 cM	15 cM	21 cM	15 cM
1-LOD boundaries	12–25 cM	117–132 cM	192–213 cM	162–177 cM
Recomb size	27 cM	13 cM	18 cM	9 cM
Recomb boundaries	0–27 cM	114–127 cM	201–219 cM	161–170 cM
No. peds (L,R bnds)	(0, 3)	(1, 1)	(2, 3)	(1, 1)
